# Supplementary material for: Rare genetic associations with human lifespan in UK Biobank are enriched for oncogenic genes
Source: Nat Commun. 2025 Feb 28;16:2064. doi: 10.1038/s41467-025-57315-6 (PMC11871019; doi:10.1038/s41467-025-57315-6)
Supplement: Supplementary file 1 — Supplementary Information [file 41467_2025_57315_MOESM1_ESM.pdf]

Rare genetic associations with human lifespan in UK Biobank are enriched for oncogenic genes

Junyoung Park[1*], Andrés Peña-Tauber[1], Lia Talozzi[1], Michael D. Greicius[1†], Yann Le Guen[2†]

Affiliations
[1]Department of Neurology and Neurological Sciences, Stanford University, Stanford, CA, 94305, USA.
[2]Quantitative Sciences Unit, Department of Medicine, Stanford University, Stanford, CA, 94304, USA.

Corresponding authors:
Junyoung Park
Stanford Neuroscience Health Center
290 Jane Stanford Way,
Stanford, CA 94305-5090
jpark01@stanford.edu
(650) 666-2696

[†]**These authors contributed equally**

Supplementary Figure 1. Phenome-wide association of rs13190937 on *ZSCAN23.* This analysis is based on PheWeb (https://pheweb.org/UKB-Neale/).

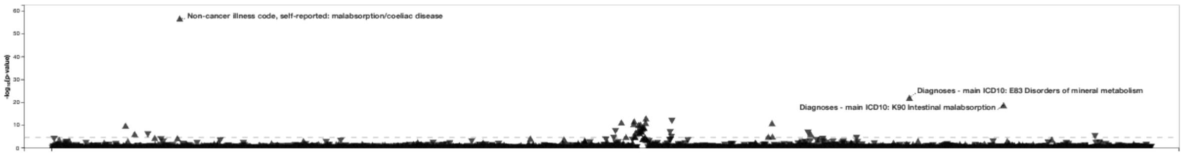

Supplementary Figure 2. Sex-stratified common variant GWAS of lifespan. Manhattan plot in males (A), and females (B). (C) Phenome-wide association of rs577106756 located in *PRKD3*. This analysis is based on PheWeb (https://pheweb.org/UKB-Neale/). Locuszoom (D) and colocalization (E) plots at the *MUC5B* locus in males, colocalized with *MUC5B* eQTL in lung tissue in GTEx. PP4: posterior probability of colocalization. (F) and (G) Phenome-wide association of rs35705950 near *MUC5B* and rs547541271 in *CELF2*, respectively, based on PheWeb (https://pheweb.org/UKB-Neale/).

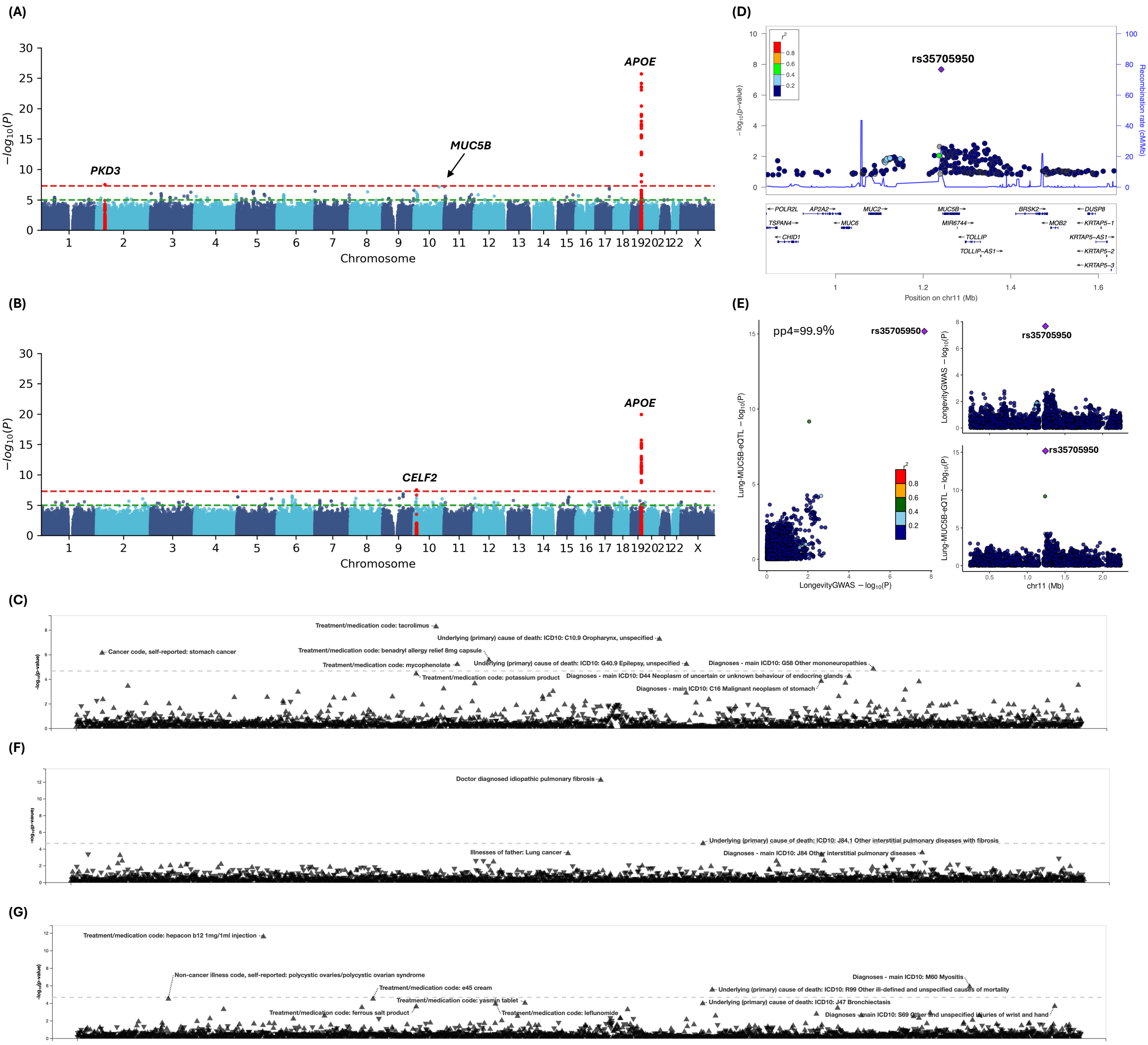

Supplementary Figure 3. Rare variant SKAT-O association with lifespan considering 3 categories: Loss-of-function (A), AlphaMissense (B), and REVEL (C). Genes highlighted in red represent those not previously identified as significant in [8]. A gene-wide significance threshold of p=7.4× 10^{-7} was applied.

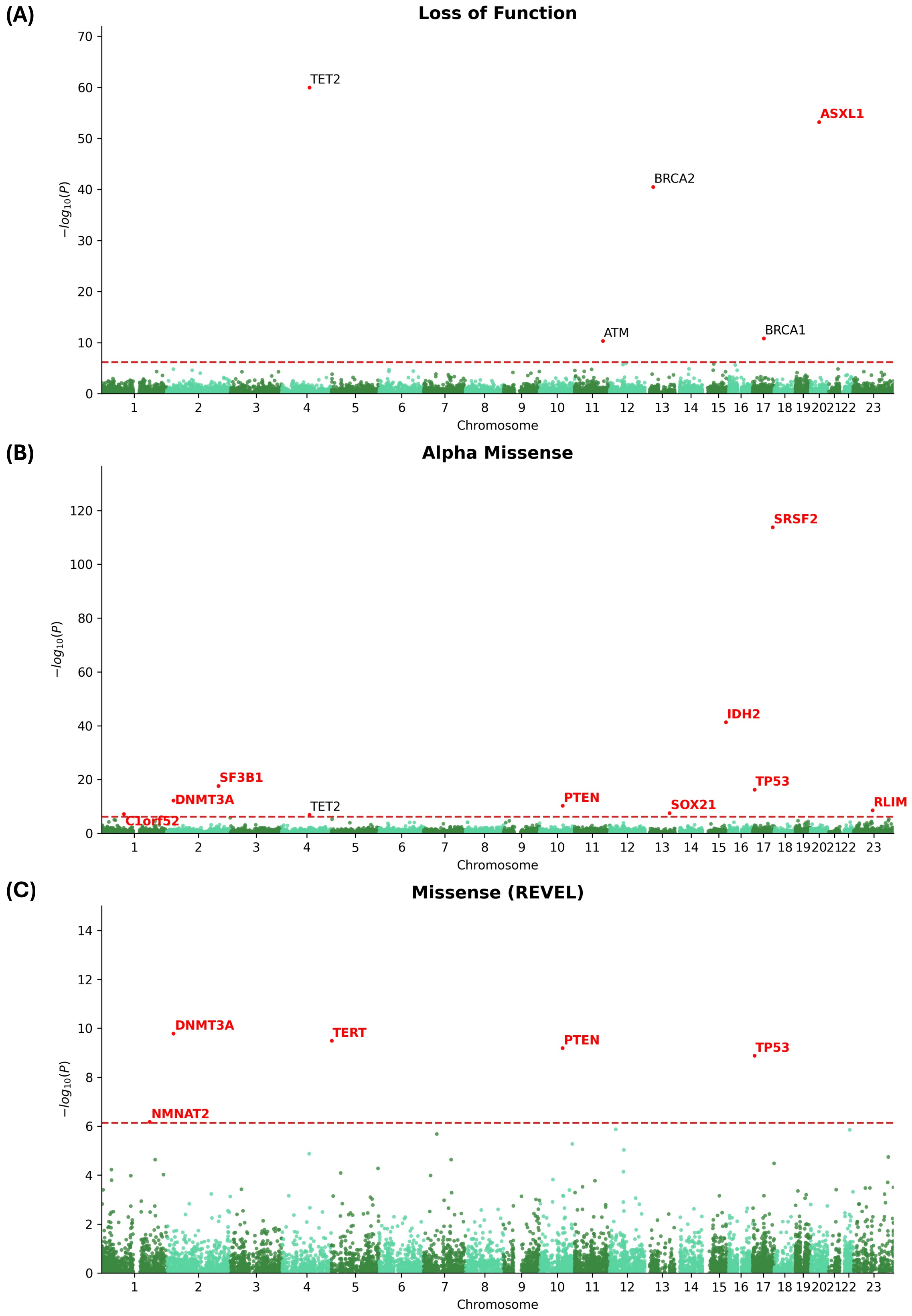

Supplementary Figure 4. Rare variant burden association with lifespan considering REVEL pathogenic missense variants. Genes highlighted in red represent those not previously identified as significant in [8]. A gene-wide significance threshold of p=7.4$\times 10^{-7}$ was applied.

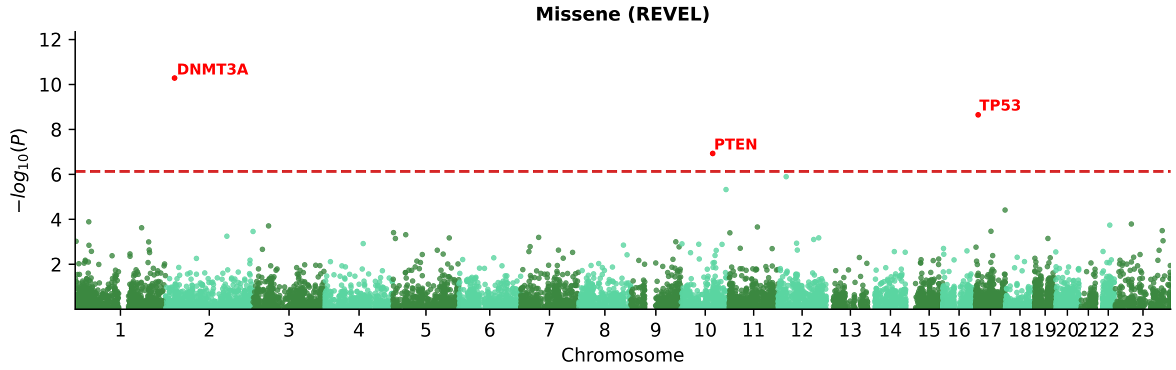

Supplementary Figure 5. Sex-stratified rare variant burden association with lifespan considering 3 categories for each sex: Loss-of-function (A), AlphaMissense (B), and REVEL (C). Genes highlighted in red represent those that were not identified as significant in the whole cohort analysis. A gene-wide significance threshold of p=7.4× 10^{-7} was applied.

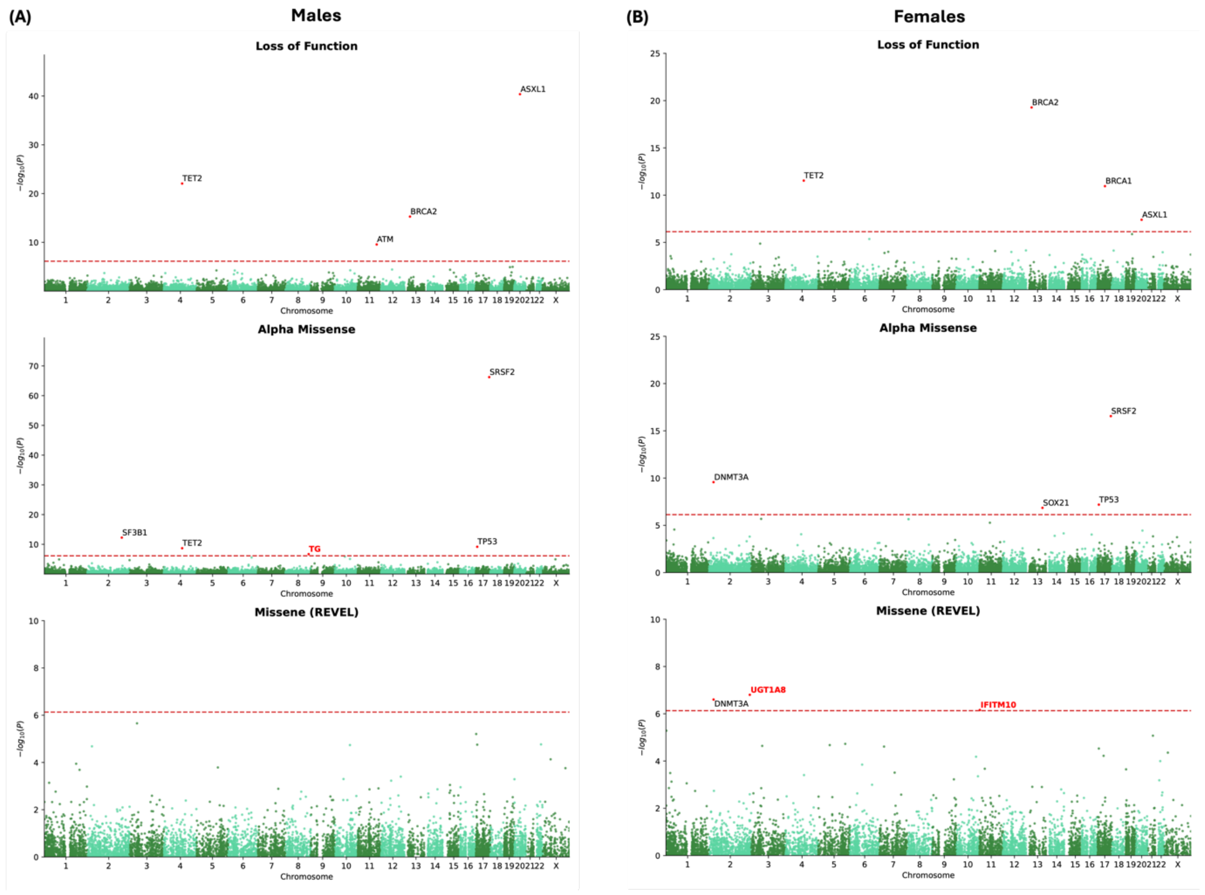

Supplementary Figure 6. Sex-stratified rare variants SKAT-O association with lifespan considering 3 categories for each sex: Loss-of-function (A), AlphaMissense (B), and REVEL (C). Genes highlighted in red represent those that were not identified as significant in the whole cohort analysis. A gene-wide significance threshold of p=7.4× 10^{-7} was applied.

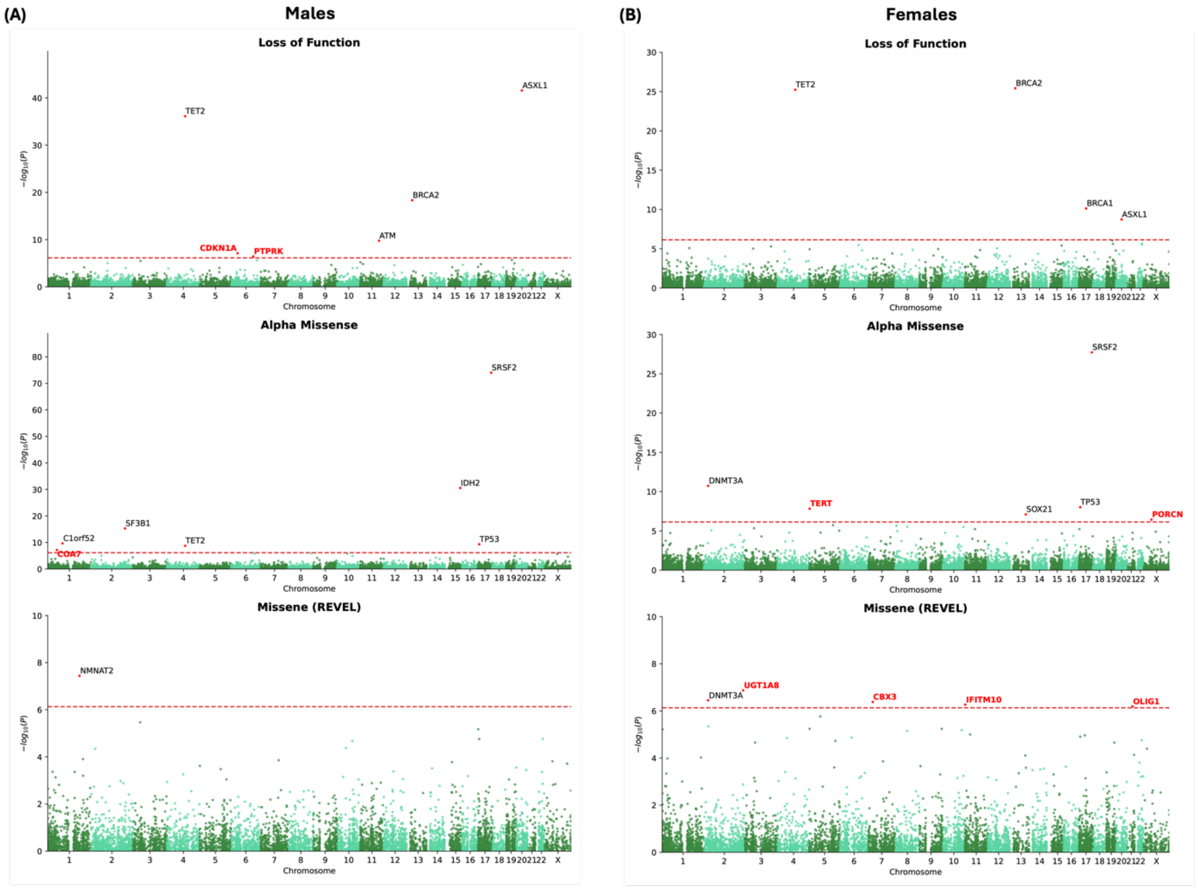

Supplementary Figure 7. Survival curves comparing carriers and non-carriers of variants considered on genes with a significant burden of loss-of-function (*TET2*, *ATM*, *BRCA2* and *BRCA1*) (A), AlphaMissense pathogenic (B) variants (*TET2*), missense variants predicted by REVEL (*DNMT3A*, *PTEN* and *TP53*) (C).

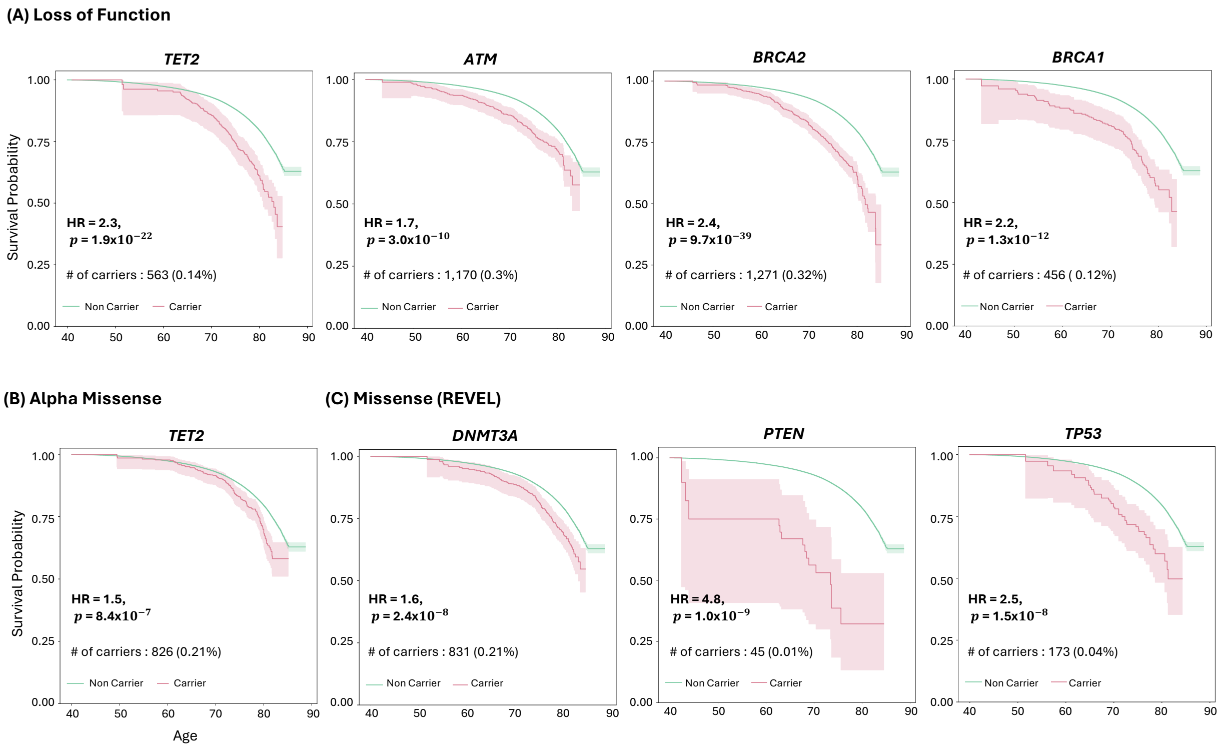

Supplementary Figure 8. Phenome-wide association of the burden of rare variants at the nine novel genes identified in our burden test. The variants include those classified as loss-of-function and those identified as AlphaMissense variants. P-values less than 1.0x10^{-50} are capped at 50.

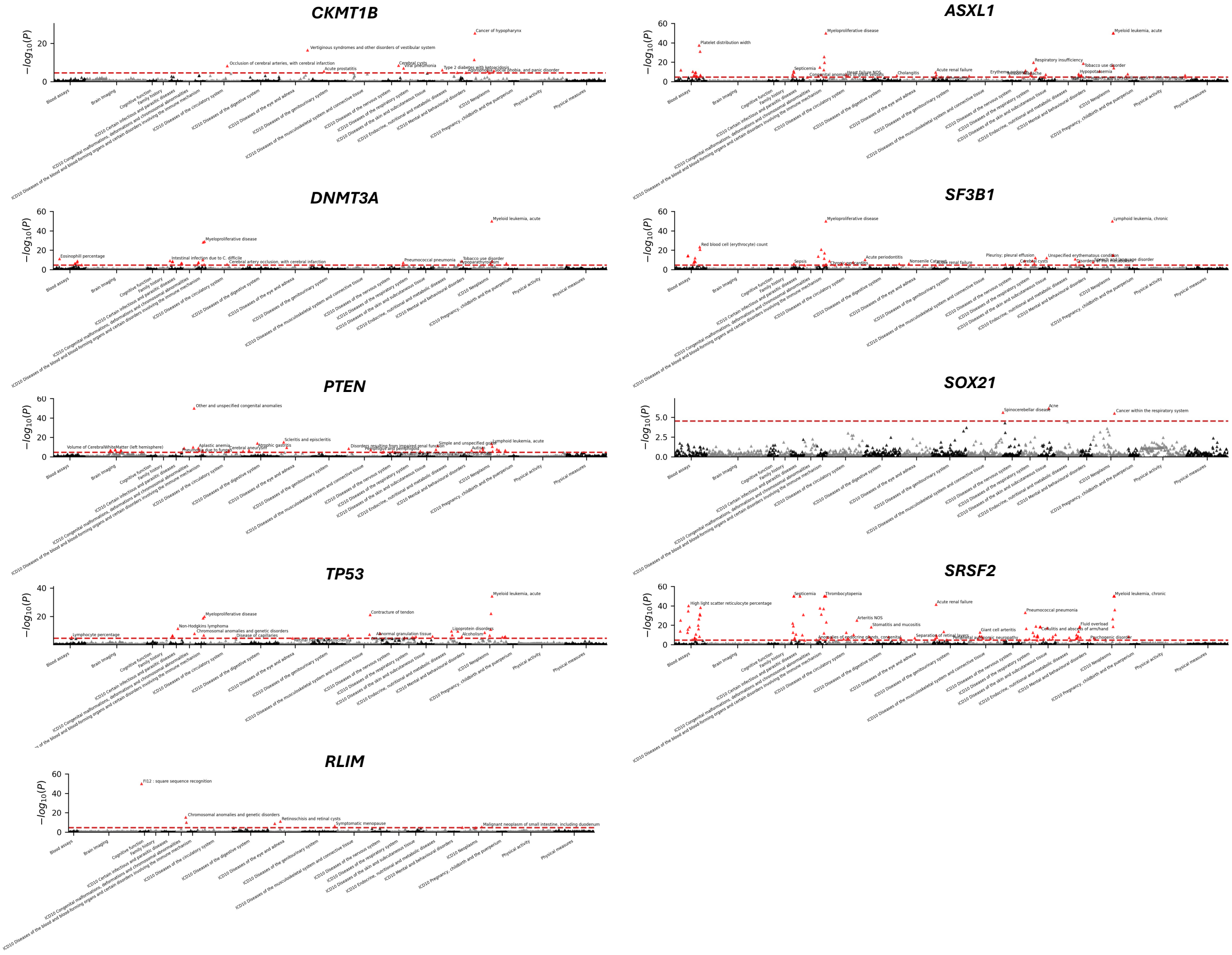

Supplementary Figure 9. Variant allelic fraction distribution per gene for variants considered in each category: Loss-of-function (A) and AlphaMissense (B).

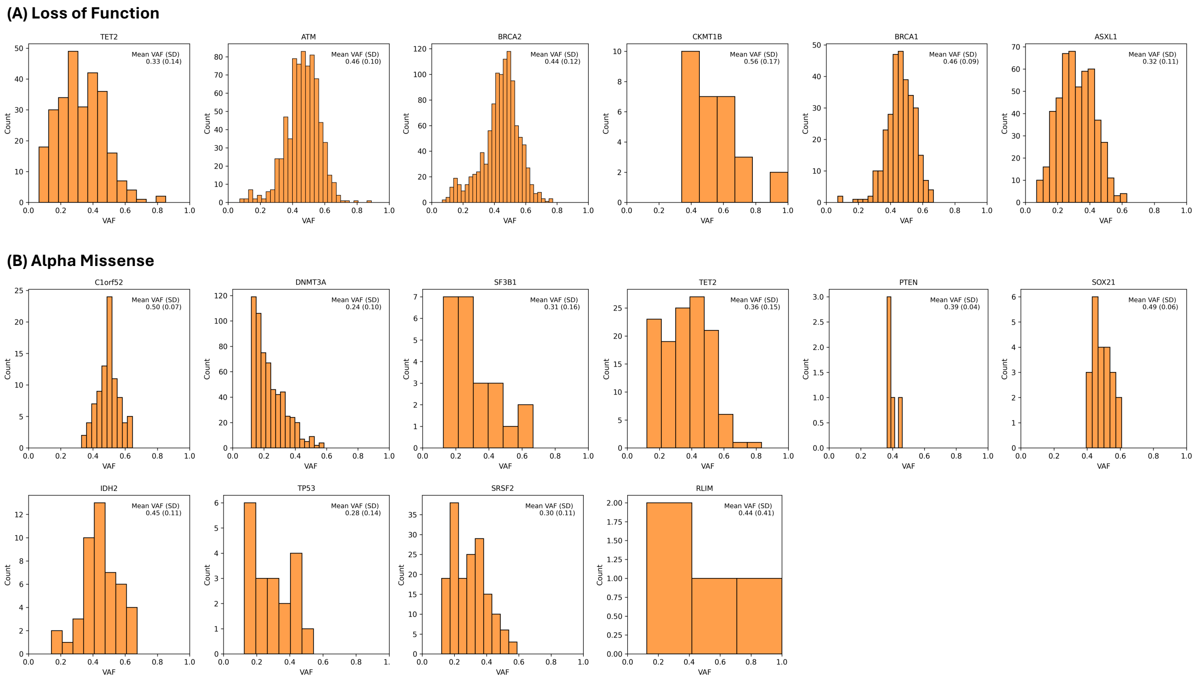

Supplementary Table 1. Demographics of European ancestry in the analyses.

	All			Male			Female		
	**Total**	**Living**	**Deceased**	**Total**	**Living**	**Deceased**	**Total**	**Living**	**Deceased**
N	393,833	358,282	35,551	180,970	159,911	21,059	212,863	198,371	14,492
Last known age	70.8 ± 7.9	70.7 ± 8.0	71.2 ± 7.5	70.9 ± 8.0	70.8 ± 8.1	71.3 ± 7.4	70.7 ± 7.9	70.7 ± 7.9	71.1 ± 7.6
APOE ε4 carrier	113,437 (28.8%)	102,360 (28.6%)	11,077 (31.2%)	52,190 (28.8%)	45,635 (28.5%)	6,555 (31.1%)	61,247 (28.8%)	56,725 (28.6%)	4,522 (31.2%)

Supplementary Table 2. Validation of Significant SNPs in FinnGen and LifeGen + UKB Cohorts for human lifespan related phenotypes.

Cohort	Phenotype	SNP	Effect allele	N	Beta	*p*-value
FinnGen (https://r11.finngen.fi)	Death	rs13190937	A	453,733	-0.011	0.20
		rs577106756	A	453,733	-0.07	0.55
		rs35705950	T	453,733	0.034	6.0×10^{-3}
		rs547541271	T	453,733	-0.018	0.93
LifeGen + UKB [2]	Parental age at death	rs13190937	A	640,183	-0.015	1.4×10^{-4}
		rs35705950	T	583,397	-0.023	6.6×10^{-3}

Supplementary Table 3. Significant genes for burden and SKAT-O association of rare variants, considering missense variants with REVEL > 75 (p<7.4$\times 10^{-7}$). Gene names in bold font represent associations identified in REVEL without significance in the LoF or AlphaMissense.

Variant Class	Chr	Gene	# of variants	# of carriers	Burden p-value	SKAT-O p-value
REVEL (>75)	1	***NMNAT2***	33	76	2.4×10^{-4}	6.7×10^{-7}
	2	*DNMT3A*	116	831	5.2×10^{-11}	1.7×10^{-10}
	5	***TERT***	31	60	3.9×10^{-4}	3.3×10^{-10}
	10	*PTEN*	42	56	1.2×10^{-7}	6.5×10^{-10}
	17	*TP53*	47	173	2.2×10^{-9}	1.3×10^{-9}

Supplementary Table 4. Gene-wide significance for rare variant burden analysis in the validation cohort. This table shows the results of the gene-wide significance tests for the 21 novel genes identified in the discovery dataset, validated in a non-British ancestry sample from the UKB (n=73,281). Ancestry distribution in the validation cohort: White (66.3%), Asian (14.4%), Black (9.9%), Other (5.7%), and Mixed (3.7%). A significance threshold of p=1.1×10^{-3} was applied after a Bonferroni correction for multiple testing. Genes that exceeded the Bonferroni correction threshold of 1.1×10^{-3} (0.05/42) are highlighted in bold font.

Variant Class	Chr	Gene	# of variants	# of carriers	Burden *p*-value	SKAT-O *p*-value
LoF	4	***TET2***	67	151	0.02	2.6×10^{-4}
	11	***ATM***	132	241	9.0×10^{-4}	2.4×10^{-4}
	13	***BRCA2***	113	218	1.1×10^{-3}	1.8×10^{-3}
	15	*CKMT1B*	11	16	0.83	1.0
	17	***BRCA1***	56	87	8.2×10^{-3}	9.2×10^{-3}
	20	***ASXL1***	28	39	1.2×10^{-5}	6.7×10^{-6}
AlphaMissense	1	*C1orf52*	14	18	0.07	0.07
	2	*DNMT3A*	59	123	0.85	0.57
	2	*SF3B1*	19	48	0.95	0.62
	4	*TET2*	50	82	0.33	0.51
	10	*PTEN*	10	33	0.79	0.43
	13	*SOX21*	21	33	0.74	0.29
	15	***IDH2***	45	65	0.73	2.0×10^{-7}
	17	*TP53*	16	23	0.10	0.06
	17	***SRSF2***	8	11	9.2×10^{-9}	2.2×10^{-10}
	X	*RLIM*	8	25	0.19	0.30
REVEL (>75)	1	*NMNAT2*	13	25	0.73	0.78
	2	*DNMT3A*	52	99	0.57	0.15
	5	*TERT*	5	7	0.55	0.74
	10	*PTEN*	8	26	0.47	0.66
	17	*TP53*	28	37	0.94	0.63

LoF: Loss of Function; Chr: chromosome

Supplementary Table 5. Significant genes for burden and SKAT-O association of rare variants in males (p<7.4× 10^{-7}). Genes in bold font represent associations that were not significant in the analysis of the entire cohort but were significant only in males.

Variant Class	Chr	Gene	# of variants	# of carriers	Burden p-value	SKAT-O p-value
LoF	4	*TET2*	162	280	8.8×10^{-23}	7.7×10^{-37}
	6	***CDKN1A***	8	33	6.0×10^{-5}	7.5×10^{-8}
	6	***PTPRK***	26	40	5.2×10^{-3}	3.7×10^{-7}
	11	*ATM*	318	520	2.7×10^{-10}	1.7×10^{-10}
	13	*BRCA2*	172	596	5.3×10^{-16}	4.7×10^{-19}
	20	*ASXL1*	59	347	4.2×10^{-41}	2.7×10^{-42}
AlphaMissense (>70)	1	*C1orf52*	19	76	1.2×10^{-5}	2.2×10^{-10}
	**1**	***COA7***	8	11	1.6×10^{-4}	6.7×10^{-8}
	2	*SF3B1*	43	122	5.5×10^{-13}	5.0×10^{-16}
	4	*TET2*	107	405	2.1×10^{-9}	1.8×10^{-9}
	**8**	***TG***	113	657	2.0×10^{-7}	1.2×10^{-6}
	15	*IDH2*	58	171	1.6×10^{-3}	2.9×10^{-31}
	17	*TP53*	24	48	6.8×10^{-10}	4.8×10^{-10}
	17	*SRSF2*	10	104	5.9×10^{-67}	9.0×10^{-75}
REVEL (>75)	1	*NMNAT2*	21	34	1.1×10^{-4}	3.6×10^{-8}

Supplementary Table 6. Significant genes for burden and SKAT-O association of rare variants in females (p<7.4× 10^{-7}). Genes in bold font represent associations that were not significant in the analysis of the entire cohort but were significant only in females.

Variant Class	Chr	Gene	# of variants	# of carriers	Burden p-value	SKAT-O p-value
LoF	4	*TET2*	151	283	2.9×10^{-12}	5.8×10^{-26}
	13	*BRCA2*	182	675	5.3×10^{-20}	3.7×10^{-26}
	17	*BRCA1*	80	217	1.1×10^{-11}	7.5×10^{-11}
	20	*ASXL1*	46	186	4.0×10^{-8}	1.9×10^{-9}
AlphaMissense (>70)	2	*DNMT3A*	135	671	2.7×10^{-10}	1.9×10^{-11}
	5	*TERT*	22	27	1.6×10^{-3}	1.5×10^{-8}
	13	*SOX21*	34	251	1.4×10^{-7}	8.1×10^{-8}
	17	*SRSF2*	10	37	2.8×10^{-17}	1.9×10^{-28}
	17	*TP53*	23	52	6.5×10^{-8}	9.7×10^{-9}
	X	***PORCN***	11	32	5.6×10^{-4}	3.7×10^{-7}
REVEL (>75)	2	*DNMT3A*	98	121	2.5×10^{-7}	3.6×10^{-7}
	2	***UGT1A8***	2	18	1.6×10^{-7}	1.3×10^{-7}
	7	***CBX3***	7	10	2.4×10^{-5}	4.2×10^{-7}
	11	***IFITM10***	10	11	7.0×10^{-7}	5.4×10^{-7}
	21	***OLIG1***	7	18	8.5×10^{-6}	6.4×10^{-7}

Supplementary Table 7. Lead variant association per gene among significant genes in the burden and SKAT-O tests. Only significant variant associations with at least 3 minor allele counts per gene are reported in this table. A significance threshold of p=8.3$\times 10^{-5}$ was applied after a Bonferroni correction for multiple testing. The "Reported" column indicates published studies that associated the highlighted variants with specific diseases, curated from ClinVar (https://www.ncbi.nlm.nih.gov/clinvar/).

**Variant Class**	**Chr**	**Gene**	**Variant**	**MA**	**MAC**	**REVEL**	**HR**	***p*-value**	**Reported**
REVEL (>75)	1	*NMNAT2*	rs201746612	A	5	0.782	11.0	1.7×10^{-6}	-
	2	*DNMT3A*	rs751562376	T	17	0.828	3.9	8.0×10^{-4}	Intellectual disability / Autism spectrum disorder [3]
	5	*TERT*	rs1043358053	C	5	0.790	16.8	1.7×10^{-8}	-
	10	*PTEN*	rs587782350	C	3	0.824	14.7	7.2×10^{-3}	Gastric Cancer [4]
	17	*TP53*	rs11540652	T	5	0.934	11.5	2.3×10^{-5}	Breast Cancer [5]

Chr: chromosome; MAC: minor allele count; REVEL: REVEL score; HR: hazard ratio

Supplementary Table 8. Mean variant allelic fraction per gene across participants included in the corresponding gene-level Burden/SKAT−O analysis.

Variant Class	Chr	Gene	# of subjects	# of variants	Mean VAF (SD)
LoF	4	*TET2*	266	133	0.33 (0.14)
	11	*ATM*	734	128	0.46 (0.10)
	13	*BRCA2*	1,061	162	0.44 (0.12)
	15	*CKMT1B*	29	8	0.56 (0.17)
	17	*BRCA1*	302	74	0.46 (0.09)
	20	*ASXL1*	502	30	0.32 (0.11)
AlphaMissense	1	*C1orf52*	87	11	0.50 (0.07)
	2	*DNMT3A*	593	33	0.24 (0.10)
	2	*SF3B1*	23	5	0.31 (0.16)
	4	*TET2*	123	41	0.36 (0.15)
	10	*PTEN*	5	3	0.39 (0.04)
	13	*SOX21*	22	6	0.49 (0.06)
	15	*IDH2*	46	14	0.45 (0.11)
	17	*TP53*	19	7	0.28 (0.14)
	17	*SRSF2*	164	6	0.30 (0.11)
	X	*RLIM*	4	2	0.44 (0.41)

Supplementary Table 9. Previously reported SNP associations with lifespan from prior studies (excluding the *APOE* region). This table summarizes SNPs previously associated with lifespan-related phenotypes in published studies that were GWAS significant in their respective studies. Notably, none of the SNPs in this table reached the suggestive threshold (p=1.0× 10^{-5}) in our analysis.

Reported phenotype	Study	Chr	Gene	Variant	p-value
Parents' attained age	Joshi et al. (2017) [6]	6	*LPA*	rs55730499	0.04
		6	*HLA-DQA1*	rs34831921	9.2×10^{-4}
		15	*CHRNA3*	rs8042849	1.5×10^{-3}
	Pilling et al. (2017) [7]	1	*CLESR2 / PSRC1 / HLA-DRB1*	rs602633	0.20
		6	*HLA-DQA1*	rs28383322	0.09
		6	*LPA*	rs55730499	0.04
		8	*EPHX2*	rs7844965	0.22
		9	*CDKN2B-AS1 (ANRIL)*	rs1556516	0.02
		12	*SH2B3 / ATXN2*	rs7137828	0.55
		14	*PROX2*	rs61978928	0.15
		15	*FURIN*	rs17514846	0.23
		15	*CHRNA3*	rs1317286	1.9×10^{-3}
	Timmers et al. (2019) [2]	1	*MAGI3*	rs1230666	0.03
		2	*KCNK3*	rs1275922	0.42
		4	*HTT*	rs61348208	0.02
		6	*HLA-DQA1*	rs34967069	1.7×10^{-3}
		6	*LPA*	rs10455872	0.03
		9	*CDKN2B-AS1*	rs1556516	0.15
		12	*ATXN2/BRAP*	rs11065979	0.88
		15	*CHRNA3/5*	rs8042849	1.5×10^{-4}
		15	*FURIN/FES*	rs6224	0.35
		16	*HP*	rs12924886	0.87
		19	*LDLR*	rs142158911	0.20

References

1. Liu JZ*, et al.* The burden of rare protein-truncating genetic variants on human lifespan. *Nature Aging* **2**, 289-294 (2022).

2. Timmers PR*, et al.* Genomics of 1 million parent lifespans implicates novel pathways and common diseases and distinguishes survival chances. *elife* **8**, e39856 (2019).

3. Yokoi T, Enomoto Y, Naruto T, Kurosawa K, Higurashi N. Tatton-Brown-Rahman syndrome with a novel DNMT3A mutation presented severe intellectual disability and autism spectrum disorder. *Hum Genome Var* **7**, 15 (2020).

4. Usui Y*, et al.* Helicobacter pylori, Homologous-Recombination Genes, and Gastric Cancer. *N Engl J Med* **388**, 1181-1190 (2023).

5. Jordan JJ*, et al.* Altered-function p53 missense mutations identified in breast cancers can have subtle effects on transactivation. *Mol Cancer Res* **8**, 701-716 (2010).

6. Joshi PK*, et al.* Genome-wide meta-analysis associates HLA-DQA1/DRB1 and LPA and lifestyle factors with human longevity. *Nature Communications* **8**, 910 (2017).

7. Pilling LC*, et al.* Human longevity: 25 genetic loci associated in 389,166 UK biobank participants. *Aging (Albany NY)* **9**, 2504-2520 (2017).
