# Supplementary material for: Rare genetic associations with human lifespan in UK Biobank are enriched for oncogenic genes
Source: Nat Commun. 2025 Feb 28;16:2064. doi: 10.1038/s41467-025-57315-6 (PMC11871019; doi:10.1038/s41467-025-57315-6)
Supplement: Supplementary file 2 — Description of Additional Supplementary Files [file 41467_2025_57315_MOESM2_ESM.pdf]

Description of Additional Supplementary Files

Supplementary Data 1. List of SNPs Used for Gene-Based Analyses. This file contains the list of SNPs included in the gene-based analyses.

Supplementary Data 2. 5-fold cross-validation Results for Gene-Based Analyses. This file contains the fold-specific results from the 5-fold cross-validation analysis conducted within the discovery dataset of 393,833 individuals. The dataset was divided into five folds, with 80% of the data (315,066 individuals) used for analysis in each fold.
