# Supplementary material for: Rare genetic associations with human lifespan in UK Biobank are enriched for oncogenic genes
Source: Nat Commun. 2025 Feb 28;16:2064. doi: 10.1038/s41467-025-57315-6 (PMC11871019; doi:10.1038/s41467-025-57315-6)
Supplement: Supplementary file 5 — Reporting Summary [file 41467_2025_57315_MOESM5_ESM.pdf]

nature portfolio

Corresponding author(s): Junyoung Park

Last updated by author(s): Jan 30, 2025

Reporting Summary

Nature Portfolio wishes to improve the reproducibility of the work that we publish. This form provides structure for consistency and transparency in reporting. For further information on Nature Portfolio policies, see our Editorial Policies and the Editorial Policy Checklist.

Statistics

For all statistical analyses, confirm that the following items are present in the figure legend, table legend, main text, or Methods section.

n/a	Confirmed	
☐	☒	The exact sample size (n) for each experimental group/condition, given as a discrete number and unit of measurement
☐	☒	A statement on whether measurements were taken from distinct samples or whether the same sample was measured repeatedly
☐	☒	The statistical test(s) used AND whether they are one- or two-sided<br>*Only common tests should be described solely by name; describe more complex techniques in the Methods section.*
☐	☒	A description of all covariates tested
☐	☒	A description of any assumptions or corrections, such as tests of normality and adjustment for multiple comparisons
☐	☒	A full description of the statistical parameters including central tendency (e.g. means) or other basic estimates (e.g. regression coefficient) AND variation (e.g. standard deviation) or associated estimates of uncertainty (e.g. confidence intervals)
☐	☒	For null hypothesis testing, the test statistic (e.g. F, t, r) with confidence intervals, effect sizes, degrees of freedom and P value noted<br>*Give P values as exact values whenever suitable.*
☒	☐	For Bayesian analysis, information on the choice of priors and Markov chain Monte Carlo settings
☒	☐	For hierarchical and complex designs, identification of the appropriate level for tests and full reporting of outcomes
☐	☒	Estimates of effect sizes (e.g. Cohen's d, Pearson's r), indicating how they were calculated

Our web collection on statistics for biologists contains articles on many of the points above.

Software and code

Policy information about availability of computer code

Data collection	No software was used to collect the data
Data analysis	We performed linear regression models using BOLT-LMM (v.2.3.4) to test the association of common variants and gene-based analyses were carried out using the SKAT package (v.2.2.5) in R (v.4.1). The codes used for analyses in the present study are available at the following link: https://github.com/Junkkkk/Lifespan-studies. The code for PheWAS analysis was utilized from https://github.com/MRCIEU/PHESANT.

For manuscripts utilizing custom algorithms or software that are central to the research but not yet described in published literature, software must be made available to editors and reviewers. We strongly encourage code deposition in a community repository (e.g. GitHub). See the Nature Portfolio guidelines for submitting code & software for further information.

Data

Policy information about availability of data

All manuscripts must include a data availability statement. This statement should provide the following information, where applicable:

- Accession codes, unique identifiers, or web links for publicly available datasets
- A description of any restrictions on data availability
- For clinical datasets or third party data, please ensure that the statement adheres to our policy

This research has been conducted using the UK Biobank database (http://www.ukbiobank.ac.uk) under application number 45420.

Research involving human participants, their data, or biological material

Policy information about studies with human participants or human data. See also policy information about sex, gender (identity/presentation), and sexual orientation and race, ethnicity and racism.

Reporting on sex and gender	We included sex as a covariate in our analysis and also performed a sex-stratified analysis.
Reporting on race, ethnicity, or other socially relevant groupings	Genetic ethnic grouping (Data Field 22006) was used to define ethnic backgrounds in this study. We restricted our analysis to subjects who were categorized as white British. In addition, we validated these findings within the UKB dataset, using an independent sample separate from our discovery data. This validation included 75,473 subjects who were not categorized as having European ancestry based on genetic ethnic grouping (Field: 21000).
Population characteristics	European Ancestry
Recruitment	The UKB database recruited participants in the United Kingdom aged 40 to 69 years between 2006 and 2010 and has since followed up with those subjects.
Ethics oversight	UKB has approval from the North West Multi-centre Research Ethics Committee (MREC) as a Research Tissue Bank (RTB) approval.

Note that full information on the approval of the study protocol must also be provided in the manuscript.

Field-specific reporting

Please select the one below that is the best fit for your research. If you are not sure, read the appropriate sections before making your selection.

☒ Life sciences ☐ Behavioural & social sciences ☐ Ecological, evolutionary & environmental sciences

For a reference copy of the document with all sections, see nature.com/documents/nr-reporting-summary-flat.pdf

Life sciences study design

All studies must disclose on these points even when the disclosure is negative.

Sample size	Total 393,833 European subjects were utilized to perform common variants GWAS and gene-based analysis for rare variants.
Data exclusions	We restricted our analysis to 393,833 individuals who self-reported their ethnic background as 'white British' and were categorized as European ancestry based on genetic ethnic grouping (Field: 21000).
Replication	In addition, we validated these findings within the UKB dataset, using an independent sample separate from our discovery data. This independent validation included 73,281 subjects who were not categorized as having European ancestry based on genetic ethnic grouping. These participants were classified into five groups based on their self-reported ethnicity (Field: 21000): White (66.3%), Asian (14.4%), Black (9.9%), Other (5.7%), and Mixed (3.7%).
Randomization	This study is not randomized.
Blinding	This is not blinding study.

Reporting for specific materials, systems and methods

We require information from authors about some types of materials, experimental systems and methods used in many studies. Here, indicate whether each material, system or method listed is relevant to your study. If you are not sure if a list item applies to your research, read the appropriate section before selecting a response.

Materials & experimental systems

n/a	Involved in the study
☒	☐ Antibodies
☒	☐ Eukaryotic cell lines
☒	☐ Palaeontology and archaeology
☒	☐ Animals and other organisms
☒	☐ Clinical data
☒	☐ Dual use research of concern
☒	☐ Plants

Methods

n/a	Involved in the study
☒	☐ ChIP-seq
☒	☐ Flow cytometry
☒	☐ MRI-based neuroimaging

Plants

Seed stocks	None
Novel plant genotypes	None
Authentication	None
